# Genomic Surveillance of Lassa Virus through In-Country Sequencing, Guinea

**DOI:** 10.3201/eid3205.260386

**Published:** 2026-05

**Authors:** Jacob Camara, Giuditta Annibaldis, Joon Klaps, Kékoura Ifono, Fara Raymond Koundouno, Youssouf Sidibé, Sarah Ryter, Moussa Conde, Saa Lucien Millimono, Mette Hinrichs, Julia Hinzmann, Nils Peter Petersen, Mia Le, Annick Renevey, Ehizojie Ehiremen Emua, Philippe Lemey, Simon Dellicour, Stephan Günther, N’Faly Magassouba, Sophie Duraffour, Liana Eleni Kafetzopoulou, Sanaba Boumbaly

**Affiliations:** Center for Virology Research—Laboratory for Viral Hemorrhagic Fevers in Guinea, Conakry, Guinea (J. Camara, M. Conde, N. Magassouba, S. Boumbaly); Bernhard Nocht Institute for Tropical Medicine, Hamburg, Germany (G. Annibaldis, S. Ryter, M. Hinrichs, J. Hinzmann, N.P. Petersen, M. Le, A. Renevey, S. Günther, S. Duraffour); German Center for Infection Research, partner site Hamburg–Lübeck–Borstel–Riems, Hamburg (G. Annibaldis, S. Ryter, M. Hinrichs, J. Hinzmann, N.P. Petersen, M. Le, A. Renevey, S. Günther, S. Duraffour); Rega Institute, KU Leuven, Leuven, Belgium (J. Klaps, P. Lemey, S. Dellicour, L.E. Kafetzopoulou); Laboratory for Viral Hemorrhagic Fevers of Guéckédou, Prefectural Health Department of Guéckédou, Guéckédou, Guinea (K. Ifono, F.R. Koundouno, S.L. Millimono); Laboratory for Viral Hemorrhagic Fevers at Regional Hospital of N’Zérékoré, Regional Hospital of N’Zérékoré, N’Zérékoré, Guinea (Y. Sidibé); University of Hamburg, Hamburg (M. Le); Irrua Specialist Teaching Hospital, Irrua, Nigeria (E.E. Emua); Spatial Epidemiology Lab, Université Libre de Bruxelles, Brussels, Belgium (S. Dellicour); Interuniversity Institute of Bioinformatics in Brussels, Université Libre de Bruxelles, Vrije Universiteit Brussel, Brussels (S. Dellicour); Leiden University Medical Center, Leiden, the Netherlands (L.E. Kafetzopoulou)

**Keywords:** viruses, zoonoses, Lassa virus, Guinea, genomic surveillance

## Abstract

Strengthened in-country sequencing generated 28 Lassa virus genomes from human clinical cases in Guinea, expanding knowledge of Lassa fever in the country. Phylogeographic analysis revealed cross-border exchange between Liberia and the N’Zérékoré region and a Sierra Leone introduction into Guéckédou. Enhanced genomic surveillance is crucial to guide public health.

Lassa fever (LF) is a life-threatening viral hemorrhagic disease endemic to West Africa; early clinical symptoms are indistinguishable from other febrile illnesses, complicating diagnosis and surveillance (*1*). The causative agent, Lassa virus (LASV), is a *Mammarenavirus* (*Arenaviridae* family) with a bisegmented (small [S] and large [L] segments) ambisense RNA genome that exhibits distinct phylogenetic structure across its endemic regions. Lineages I–III and VI circulate in Nigeria, lineage IV predominates in the Mano River Union countries (Guinea, Sierra Leone, and Liberia), and additional distinct lineages circulate in Mali/Côte d’Ivoire (lineage V) and Togo (lineage VII). Although LF cases are only sporadically reported in Guinea, serologic evidence from the southeastern (forested) and central regions indicates broad population exposure (*2*,*3*). Sequencing efforts thus far have generated partial LASV genomes from rodent reservoirs in Upper Guinea (*4*), whereas genomes from human infections remain limited (*5*,*6*). Sparse genomic data limit our understanding of geogenomic variation, outbreak dynamics, and clinical correlations, highlighting the need for enhanced genomic surveillance to inform diagnostics, epidemiology, and patient management. 

To expand viral surveillance and diagnostic capabilities locally in Guinea, genomic capacity strengthening was initiated in 2021 at the Centre de recherche en Virologie–Laboratoire des Fièvres Hémorragiques Virales de Guinée (CRV-LFHVG; Conakry, Guinea). Sequencing infrastructure was initially established in response to the COVID-19 pandemic using targeted Nanopore sequencing (Oxford Nanopore Technologies, https://nanoporetech.com) (*7*). In 2022–2023, laboratory capacity was expanded to include metagenomic sequencing (*8*,*9*), integrated within the diagnostic network of 3 surveillance laboratories for viral hemorrhagic fevers across Guinea: CRV-LFHVG, the national reference laboratory in Conakry, and 2 satellite laboratories in the forest region, in Guéckédou (Laboratoire des Fièvres Hémorragiques Virales de Guéckédou; LFHV-GKD) and N’Zérékoré (Laboratoire des Fièvres Hémorragiques Virales de Hôpital Régional de N’Zérékoré; LFHV-HRNZE). During 2020–2024, this laboratory network confirmed a total of 36 LF cases (F.R. Koundouno et al., unpub. data, https://www.medrxiv.org/content/10.64898/2026.02.24.26346968v1), including a nosocomial outbreak in Conakry in 2022 (*10*). In-country metagenomic nanopore sequencing was performed at CRV-LFHVG to investigate the viral diversity of the confirmed LF cases. A total of 28 LF cases were successfully sequenced, all of which yielded sufficient genomic coverage for downstream phylogenetic analysis (Appendix Table). Most sequencing yielded near-complete genomes for both segments (Appendix Table), and all sequences were phylogenetically classified as lineage IV.

Phylogenetic analysis revealed that many of the newly sequenced genomes from Guinea are substantially divergent from previously characterized cases; branch lengths suggest years to decades of virus circulation in the natural reservoir before sampling in human cases (Figures 1, 2). Bayesian phylogeographic reconstruction estimated substitution mean rates of 8.5 × 10^−4^ (S segment) and 8.2 × 10^−4^ (L segment) substitutions/site/year, placing the root of lineage IV in the 17th–18th Centuries, likely originating in southeastern (forested) Guinea (Figures 1, 2).

**Figure 1:**
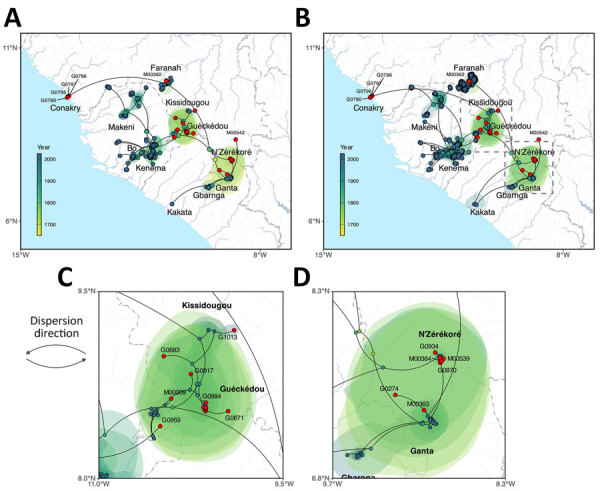
Phylogeographic reconstruction of the dispersal history of Lassa virus lineage IV from study of genomic surveillance of Lassa virus through in-country sequencing, Guinea. A, B) Results of the continuous phylogeographic inference based on large (A) and small (B) segment sequences. For each analysis, the corresponding maximum clade credibility tree is mapped with internal and tip nodes colored according to their estimated time of occurrence and sampling date. Tip nodes corresponding to newly sequenced cases from this study are highlighted in red. Shaded polygons represent the 80% highest posterior density regions, reflecting uncertainty in internal node location inference. The estimated root of lineage IV, dating to the 17th–18th Centuries, is indicated by dashed squares in the southeastern (forested) region of Guinea. C, D) Maps highlighting specific transmission dynamics in Guéckédou (C) and N’Zérékoré (D), illustrating the dense local clustering and the cross-border introductions from Liberia (Ganta) into the N’Zérékoré region.

**Figure 2:**
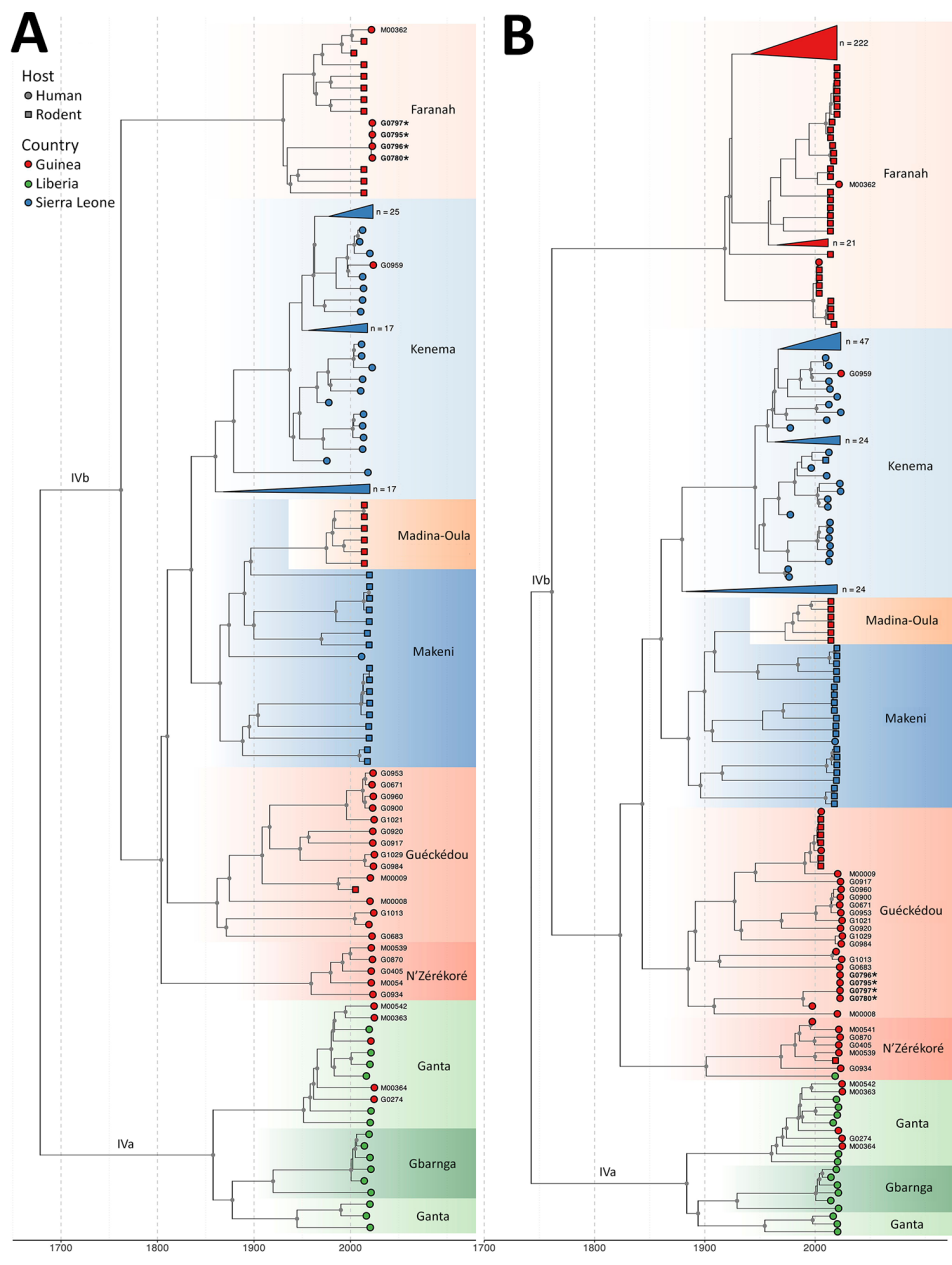
Temporal evolution of the large and small segments of Lassa virus from study of genomic surveillance of Lassa virus through in-country sequencing, Guinea. Time-scaled maximum clade credibility trees are shown for the large (A) and small (B) segments. Tips are colored by country of origin. Sublineages are annotated and colored by their predominant geographic location (e.g., Kenema, Faranah, Ganta). Clades that fall outside the sequence diversity of interest (i.e., clades that have no association with the sequences reported in this article) are collapsed and annotated with the total number of sequences they include. Gray dots indicate internal nodes with a clade credibility of >80%. Sequences reported in this manuscript have their sample identification codes indicated next to their respective tip. We detected 2 cocirculating sublineages of Lassa virus in N’Zérékoré: a locally established IVb lineage (M00539, M00541, G0405, G0870, G0934), and the IVa lineage linked to the region of Ganta in Liberia (M00363, M00542, M00364, G0274). The sample from Guéckédou (G0959) grouped within the Kenema, Sierra Leone, Lassa virus cluster, reflecting its travel-linked origin. Strains that form the nosocomial transmission chain with reassorted genomes have been highlighted in bold and are indicated with a star.

Spatial viral diversity in Guinea is organized into 3 predominant geographic clusters, mainly associated with the areas of Guéckédou, N’Zérékoré, and Faranah. That factor should be interpreted cautiously, because Guéckédou and N’Zérékoré both have surveillance laboratories, and sequences from the Faranah region originate from rodent reservoirs sampled during previous studies. Consequently, the inferred geographic clustering might be driven, at least in part, by uneven sampling. Two cocirculating sublineages of LASV were identified in the N’Zérékoré region: an older IVb lineage, which has been long-established locally (M00539, M00541, G0405, G0870, G0934); and the IVa lineage, which traces back to multiple independent introductions (M00363, M00542, M00364, G0274) from Ganta, northeastern Liberia, with common ancestors dating to the 1950s–1980s (Figure 1, panel D; Figure 2). In Guéckédou, we detected an imported LASV case originating from the Kenema area of Sierra Leone (G0959) (Figure 1, panel C; Figure 2), illustrating that sporadic introductions from neighboring LASV-endemic regions to Guinea can occur and contribute to Guinean LASV diversity.

We included 4 LF cases from Conakry, in the western part of Guinea, all of which were associated to a previously reported nosocomial transmission chain linked to a travel case from a LASV-endemic area (*10*) (Appendix). The LASV sequences (G0780, G0795, G0796, G0797) show minimal between-sequence variation (L segment, 0–4 mutations; S segment, 0–1 mutations), consistent with a single transmission event. We identified all 4 genomes as a reassortant LASV variant with their 2 segments clustering significantly differently (Appendix). Their L segments clustered with sequences previously identified in Faranah (lineage IVb in Figure 2, panel A), and their S segments clustered with sequences previously identified in Guéckédou (lineage IVb in Figure 2, panel B).

This study increases the available LASV sequences derived from human clinical cases and provides new genomic insights into LASV circulation in Guinea. Our findings were made possible through strengthened laboratory diagnostics in LF-endemic areas (Guéckédou and N’Zérékoré) and the establishment of new sequencing capacity for viral hemorrhagic fevers at CRV-LFHVG, Conakry. Ongoing genomic surveillance remains crucial for guiding public health interventions, as well as for the development of appropriate medical countermeasures.

Appendix Additional information about genomic surveillance of Lassa virus through in-country sequencing, Guinea
