# Supplementary material for: Genomic Surveillance of Lassa Virus through In-Country Sequencing, Guinea
Source: Emerg Infect Dis. 2026 May;32(5):819–24. doi: 10.3201/eid3205.260386 (PMC13175098; doi:10.3201/eid3205.260386)
Supplement: Appendix — Additional information about genomic surveillance of Lassa virus through in-country sequencing, Guinea [file 26-0386-Techapp-s1.pdf]

Article DOI: https://doi.org/10.3201/eid3205.260386

EID cannot ensure accessibility for supplementary materials supplied by authors. Readers who have difficulty accessing supplementary content should contact the authors for assistance.

Genomic Surveillance of Lassa Virus through In-Country Sequencing, Guinea

Appendix

Materials and methods

Laboratory investigations

EDTA-blood was collected from VHFs suspected cases. Real-time reverse transcription polymerase chain reaction (RT-PCR) was performed at the *Centre de recherche en Virologie - Laboratoire des Fièvres Hémorragiques Virales de Guinée* (CRV-LFHVG), the *Laboratoire des Fièvres Hémorragiques Virales de Guéckédou* (LFHV-GKD) and *Laboratoire des Fièvres Hémorragiques Virales de Hôpital Régional de N'Zérékoré* (LFHV-HRNZE). The detailed description of laboratory surveillance for Lassa fever in Guinea and Lassa cases is provided elsewhere (*1*) (F.R. Koundouno, unpub. data, https://www.medrxiv.org/content/10.64898/2026.02.24.26346968v1). Viral RNA was extracted with the QIAamp viral RNA extraction kit (Qiagen, Germany) using 70 µl of the human plasma (or serum) together with 70 µl of nuclease-free water and processed according to the manufacturer's instructions with the addition of two rounds of buffer AW2 washes and a 10-minute dry spin. RNA extracts were used for RT-PCR on the Rotor-Gene Q platform (Qiagen). Leftover RNAs were stored at −20°C. For Lassa virus diagnostics, the RealStar® Lassa Virus RT-PCR Kit 2.0 from altona Diagnostics (Germany) was used. A Lassa virus positive RT-PCR result is defined as at least one of the two targets (S or L-segment) being detected as per manufacturer instructions.

Metagenomic sequencing

Leftover RNA extracts or newly extracted RNAs were used for nanopore next generation sequencing on the MinION platform (Oxford Nanopore Technologies (ONT), United Kingdom). RNA extracts and sequencing libraries were prepared as described previously at CRV-LFHVG (*2–4*). Briefly, viral RNA was digested with DNase (TURBO

DNase, Thermo Fisher Scientific) and then randomly reverse-transcribed, and amplified using a Sequence Independent Single Primer Amplification (SISPA) approach. MinION sequencing libraries were prepared using the Ligation Sequencing Kit (SQK-LSK109) according to manufacturer's instructions. Libraries were loaded onto the R9.4.1 Flow Cells (FLO-MIN106D, ONT) and run on the Mk1C (ONT) device. Sequencing flow cells were reloaded with leftover libraries after 24 hr. Runs were further stopped after ~48 hr and fast5 files were transferred to a laptop for basecalling and demultiplexing. Before 2025, a fastq files were generated from the fast5 files using Guppy v5.0.16, and consensus genomes were obtained using minimap2 v.2.17 and CANU v1.9 (*3*). For this work, consensus genomes were re-generated using Dorado v0.7.2 and the upgraded metagenomic nanopore pipeline ViMOP (*5*). The majority consensus sequence consisted of bases called at a minimum depth of 20x and 70% base predominance per nucleotide location. The complete sequences have been submitted to GenBank (GenBank IDs: PV847661–66; PX115263- PX115312) (Appendix Table). Sequences from cases associated with the nosocomial outbreak in 2022 in Guinea (GenBank IDs: PV847661–66) have been obtained and described elsewhere (*1*).

Phylogenetic analysis

All publicly available Lassa virus (LASV) sequences were downloaded from NCBI Virus GenBank on July 25, 2025 (*Mammarenavirus lassaense* species, taxid:3052310). Sequence deduplication was performed using MMseqs2 v14.7e284 (*6*) to identify duplicates based on high sequence similarity (≥99.9% identity based on high sequence similarity (≥99.9% identity and <2 mismatches). Sequences shorter than 500 bp, sequences associated with patents or vaccines, and sequences lacking sufficient geographic precision were excluded. Only sequences from Guinea, Sierra Leone and Liberia were kept. Genes were extracted and aligned to reference sequences using MAFFT v7.508 (*7*), then concatenated in a consistent orientation allowing codon partitioning. To identify potential outliers and validate the alignment, a maximum likelihood tree was constructed using IQ-TREE v2.1.4 (*8*). Misaligned regions were manually inspected and corrected.

Time-calibrated phylogenies were estimated in BEAST X v10.5.0 (*9*), with a chain of 2 × 500 billion interactions using a GTR+Γ4 substitution model with codon partitions, an uncorrelated lognormal relaxed molecular clock, and a Bayesian skygrid coalescent prior for population dynamics. Spatial diffusion was modeled with a Relaxed Random Walk model. Logs were combined after discarding the first 10 million iterations of each run as burn-in, and posterior trees were subsequently summarized as maximum clade credibility (MCC) trees

using TreeAnnotater v10.5.0 (*9*). Visualization of the spatiotemporal dispersal history was performed using the `ggphylogeo` v.0.1.2 and `ggtree` v4.0.4 R packages (*9*,*10*). Reassortment analysis of the concatenated L and S segments in RDP4 (*11*), using a full exploratory recombination scan, identified samples G0796, G0797, and G0795 as significant recombinants. These events were strongly supported by five out of nine algorithms: Bootscan (p = 1.65E-03), Maxchi (p = 4.70E-12), Chimaera (p = 3.33E-07), SiSscan (p = 4.42E-50) and 3Seq (p = 2.83E-19), but were not significant by RDP, GENECONV, PhyloPro and LARD (*12*–*20*).

References

1. Annibaldis G, Soropogui B, Ifono K, Camara J, Kaba ML, Berete F, et al. Nosocomial outbreak of Lassa fever in Conakry, Guinea, 2022. J Infect Dis. 2026. In press.

2. Kafetzopoulou LE, Efthymiadis K, Lewandowski K, Crook A, Carter D, Osborne J, et al. Assessment of metagenomic Nanopore and Illumina sequencing for recovering whole genome sequences of chikungunya and dengue viruses directly from clinical samples. Euro Surveill. 2018;23:1800228. PubMed https://doi.org/10.2807/1560-7917.ES.2018.23.50.1800228

3. Kafetzopoulou LE, Pullan ST, Lemey P, Suchard MA, Ehichioya DU, Pahlmann M, et al. Metagenomic sequencing at the epicenter of the Nigeria 2018 Lassa fever outbreak. Science. 2019;363:74–7. PubMed https://doi.org/10.1126/science.aau9343

4. Koundouno FR, Kafetzopoulou LE, Faye M, Renevey A, Soropogui B, Ifono K, et al. Detection of Marburg virus disease in Guinea. N Engl J Med. 2022;386:2528–30. PubMed https://doi.org/10.1056/NEJMc2120183

5. Petersen NP, Le M, Renevey A, Emua E, Ryter S, Annibaldis G, et al. ViMOP: a user-friendly and field-applicable pipeline for untargeted viral genome nanopore sequencing. Bioinformatics. 2026;42:btaf687. PubMed https://doi.org/10.1093/bioinformatics/btaf687

6. Steinegger M, Söding J. MMseqs2 enables sensitive protein sequence searching for the analysis of massive data sets. Nat Biotechnol. 2017;35:1026–8. PubMed https://doi.org/10.1038/nbt.3988

7. Katoh K, Standley DM. MAFFT multiple sequence alignment software version 7: improvements in performance and usability. Mol Biol Evol. 2013;30:772–80. PubMed https://doi.org/10.1093/molbev/mst010

8. Minh BQ, Schmidt HA, Chernomor O, Schrempf D, Woodhams MD, von Haeseler A, et al. IQ-TREE 2: new models and efficient methods for phylogenetic inference in the genomic era. Mol Biol Evol. 2020;37:1530–4. PubMed https://doi.org/10.1093/molbev/msaa015

9. Baele G, Ji X, Hassler GW, McCrone JT, Shao Y, Zhang Z, et al. BEAST X for Bayesian phylogenetic, phylogeographic and phylodynamic inference. Nat Methods. 2025;22:1653–6. PubMed https://doi.org/10.1038/s41592-025-02751-x

10. Klaps J, Brusselmans MD, Dellicour S, Kafetzopoulou LE, Lemey P. Joon-Klaps/ggphylogeo: version 0.1.2 [cited 2026 Feb 6]. https://zenodo.org/records/18507142.

11. Martin DP, Varsani A, Roumagnac P, Botha G, Maslamoney S, Schwab T, et al. RDP5: a computer program for analyzing recombination in, and removing signals of recombination from, nucleotide sequence datasets. Virus Evol. 2020;7:veaa087. PubMed https://doi.org/10.1093/ve/veaa087

12. Weiller GF. Phylogenetic profiles: a graphical method for detecting genetic recombinations in homologous sequences. Mol Biol Evol. 1998;15:326–35. PubMed https://doi.org/10.1093/oxfordjournals.molbev.a025929

13. Smith JM. Analyzing the mosaic structure of genes. J Mol Evol. 1992;34:126–9. PubMed https://doi.org/10.1007/BF00182389

14. Posada D, Crandall KA. Evaluation of methods for detecting recombination from DNA sequences: computer simulations. Proc Natl Acad Sci U S A. 2001;98:13757–62. PubMed https://doi.org/10.1073/pnas.241370698

15. Padidam M, Sawyer S, Fauquet CM. Possible emergence of new geminiviruses by frequent recombination. Virology. 1999;265:218–25. PubMed https://doi.org/10.1006/viro.1999.0056

16. Martin DP, Posada D, Crandall KA, Williamson C. A modified bootscan algorithm for automated identification of recombinant sequences and recombination breakpoints. AIDS Res Hum Retroviruses. 2005;21:98–102. PubMed https://doi.org/10.1089/aid.2005.21.98

17. Martin D, Rybicki E. RDP: detection of recombination amongst aligned sequences. Bioinformatics. 2000;16:562–3. PubMed https://doi.org/10.1093/bioinformatics/16.6.562

18. Lam HM, Ratmann O, Boni MF. Improved algorithmic complexity for the 3SEQ recombination detection algorithm. Mol Biol Evol. 2018;35:247–51. PubMed https://doi.org/10.1093/molbev/msx263

19. Holmes EC, Worobey M, Rambaut A. Phylogenetic evidence for recombination in dengue virus. Mol Biol Evol. 1999;16:405–9. PubMed https://doi.org/10.1093/oxfordjournals.molbev.a026121

20. Gibbs MJ, Armstrong JS, Gibbs AJ. Sister-scanning: a Monte Carlo procedure for assessing signals in recombinant sequences. Bioinformatics. 2000;16:573–82. PubMed https://doi.org/10.1093/bioinformatics/16.7.573

Appendix Table. Details of 28 Lassa fever cases with successful sequencing results

Nr	ID	Age, y/sex	Location	Date-sampling	Ct value S diag	Ct value L diag	Coverage S [%]	Coverage L [%]	GenBank ID S	GenBank ID L
1	M00008	56/M	Guéckédou	12-Mar-2020	36.2	31.3	72.9	60.8	PX115298	PX115297
2	M00009	28/F	Guéckédou	10-Jul-2020	25.6	21.7	99.8	99.6	PX115300	PX115299
3	G0274	25/M	Yomou	7-May-2021	29.6	29.5	91.6	84.6	PX115264	PX115263
4	M00542*	50/M	Beyla	12-May-2021	40.4	33.4	77.9	62.9	PX115311	PX115312
5	G0405	65/F	N'Zérékoré	15-Jun-2021	28.7	26.5	99.3	99.4	PX115266	PX115265
6	M00541	52/M	N'Zérékoré	29-Jun-2021	31.2	34.8	88.6	88.9	PX115310	PX115309
7	M00539	12/F	N'Zérékoré	14-Jul-2021	27.3	45.0	99..8	99..3	PX115307	PX115308
8	M00363	30/M	Yomou	17-Aug-2021	23.4	22.0	99.8	99.56	PX115304	PX115303
9	M00364	40/F	N'Zérékoré	20-Aug-2021	32.7	26.5	96.0	98.1	PX115306	PX115305
10	M00362	9/F	Faranah	18-Sep-2021	21.3	19.3	99.4	99.7	PX115302	PX115301
11	G0683	17/F	Guéckédou	20-Apr-2022	33.9	30.5	62.2	69.8	PX115270	PX115269
12	G0671	24/M	Guéckédou	28-Apr-2022	32.3	29.0	70.6	70.9	PX115267	PX115268
13	G0780†	27/F	Conakry	9-Aug-2022	30.0	31.2	35.7	42.3	PX115272	PX115271
14	G0795†	30/F	Conakry	10-Aug-2022	26.0	23.5	95.5	94.7	PV847661	PV847662
15	G0796†	25/M	Conakry	10-Aug-2022	23.3	21.0	98.3	99.7	PV847663	PV847664
16	G0797†	46/M	Conakry	11-Aug-2022	21.4	23.7	95.6	99.4	PV847665	PV847666
17	G0870	48/M	N'Zérékoré	19-Sep-2022	31.6	28.1	43.3	67.0	PX115273	PX115274
18	G0900	75/F	Guéckédou	7-Dec-2022	21.7	23.5	99.8	99.6	PX115276	PX115275
19	G0917	35/F	Guéckédou	25-Jan-2023	17.7	18.0	99.9	99.6	PX115278	PX115277
20	G0920	37/M	Guéckédou	25-Jan-2023	30.6	27.4	72.2	62.3	PX115279	PX115280
21	G0934	3/M	N'Zérékoré	21-Mar-2023	28.0	24.2	98.2	43.7	PX115282	PX115281
22	G0953	20/M	Guéckédou	9-May-2023	23.7	19.3	99.6	99.6	PX115283	PX115284
23	G0959	22/M	Guéckédou	31-Jul-2023	30.6	20.2	99.6	95.4	PX115286	PX115285
24	G0960	27/M	Guéckédou	4-Aug-2023	45.0	25.9	96.0	93.3	PX115287	PX115288
25	G0984	39/M	Guéckédou	26-Sep-2023	22.6	24.7	99.6	99.5	PX115289	PX115290
26	G1013	27/F	Kissidougou	31-Jan-2024	18.4	19.3	94.5	97.7	PX115292	PX115291
27	G1021	23/M	Guéckédou	11-Jun-2024	20.5	19.5	98.9	98.6	PX115294	PX115293
28	G1029	63/M	Guéckédou	21-Sep-2024	14.5	16.2	99.5	97.3	PX115296	PX115295

*Confirmed LF case detected after retrospective testing.
†Cases from the nosocomial LF outbreak in Conakry, 2022.
